# Extrapulmonary Small Cell Carcinoma of the Seminal Vesicles and Prostate Demonstrated on 18F-FDG Positron Emission Tomography/Computed Tomography

**DOI:** 10.4274/mirt.02997

**Published:** 2016-02-10

**Authors:** Amir Iravani Tabrizipour, Lily Shen, Robert Mansberg, Bui Chuong

**Affiliations:** 1 Nepean Hospital, Department of Nuclear Medicine and PET, Penrith, Australia; 2 Sydney University Sydney Medical School, NSW, Australia

**Keywords:** Fluorine-18-fluorodeoxyglucose, Positron-emission tomography, small cell carcinoma, seminal vesicles, prostate cancer

## Abstract

Extrapulmonary primary small cell carcinomas arising from the urogenital tract is infrequent. It can rarely arise from the prostate and even more rarely from the seminal vesicles. We present a 79-year-old male who was admitted due to acute renal failure with a history of radical radiotherapy for prostate adenocarcinoma 13 years ago. The prostate specific antigen level was not elevated. An abdominopelvic computed tomography (CT) scan showed markedly enlarged seminal vesicles causing bilateral ureteral obstruction and a mildly enlarged prostate. Further evaluation with fluorine-18-fluorodeoxyglucose (^0^F-FDG) positron emission tomography/CT demonstrated extensive ^18^F-FDG uptake in the pelvis with diffuse involvement of both seminal vesicles and the prostate without pathologic uptake in the lungs or elsewhere in the body. Core biopsies of the prostate and both seminal vesicles revealed diffuse involvement by small cell carcinoma. Therapy could not be instituted due to a rapid deterioration in the patient’s clinical condition.

## Abstract (tr)

Ürogenital sistem kaynaklı ekstrapulmoner primer küçük hücreli karsinom nadir görülür. Seyrek olarak prostattan daha da nadir olarak seminal veziküllerden kaynaklanabilir. Bu yazıda, 13 yıl önce prostat adenokarsinomu nedeniyle radikal radyoterapi uygulanma öyküsü bulunan ve akut böbrek yetmezliği nedeniyle yatırılan 79 yaşında bir erkek hasta sunulmaktadır. Prostat spesifik antijen değeri yükselmemişti. Abdominopelvik bilgisayarlı tomografide (BT) bilateral üreter obstrüksiyonuna neden olacak şekilde ciddi derecede büyümüş seminal veziküller ve hafif büyümüş prostat bezi saptandı. Fluorodeoksiglukoz F18 (^18^F-FDG) pozitron emisyon tomografi/BT pelviste her iki seminal vezikül ve prostatta yaygın ^18^F-FDG tutulumu saptadı, akciğerler ya da diğer vücut bölgelerinde patolojik tutulum saptanmadı. Prostat ve her iki seminal vezikülden yapılan kor biyopsiler yaygın küçük hücreli karsinom ortaya koydu. Hastanın klinik durumunda hızla kötüleşme nedeniyle tedaviye başlanamadı.

## INTRODUCTION

Small-cell carcinoma usually originates from the lung and accounts for 18% of all lung cancers. Nevertheless, the primary site can rarely be outside the lungs and pleural spaces, which are referred to as extrapulmonary small-cell carcinoma (EPSCC) (1). Besides the respiratory tract, small cell carcinoma can rarely arise from the prostate and in extremely rare cases from the seminal vesicles (2). Herein, we present a rare case of EPSCC demonstrated on fluorine-18-fluorodeoxyglucose (^18^F-FDG) positron emission tomography/computed tomography (PET/CT) with extensive involvement of the seminal vesicles and prostate gland.

## CASE REPORT

**Figure 1:**
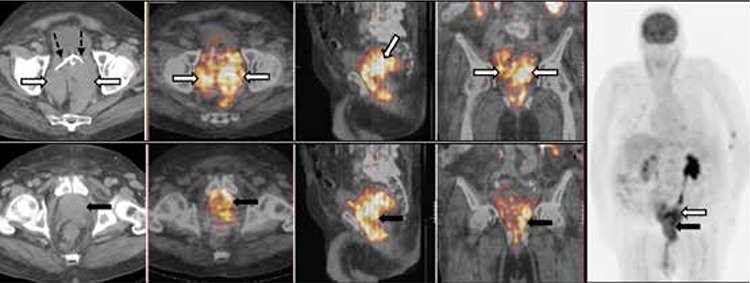
Upper row: Axial pelvic computed tomography demonstrated bilateral markedly enlarged seminal vesicles occupying most of the pelvic cavity, measuring 9x6 cm on the left and 7x4 cm on the right (white arrows), causing bilateral ureteral obstruction requiring bilateral ureteral stents (dashed arrows). Fusion fluorine-18-fluorodeoxyglucose (^18^F-FDG) positron emission tomography/computed tomography (PET/CT) (axial, sagittal and coronal) demonstrated heterogeneous intense ^18^F-FDG uptake (SUV_max_: 7.2) throughout the markedly enlarged seminal vesicles (white arrows). The patient had an indwelling urinary catheter, hence no significant retained urinary tracer activity was seen in the bladder and no obvious abnormal tracer activity was seen in the bladder wall, Lower row: Axial pelvic CT demonstrated mildly enlarged prostate gland. Fusion ^18^F-FDG PET/CT (axial, sagittal and coronal) demonstrated diffusely increased ^18^F-FDG uptake in the prostate gland with direct invasion of the pelvic floor not excluded (black arrows), Anterior whole body MIPs image demonstrated no suspicious focal FDG uptake outside the pelvis to suggest a primary lesion elsewhere (including the lungs) or metastatic spread. Low-grade focal uptake in the anterior ends of the right 3^rd^-5^th^ ribs was consistent with prior trauma

**Figure 2:**
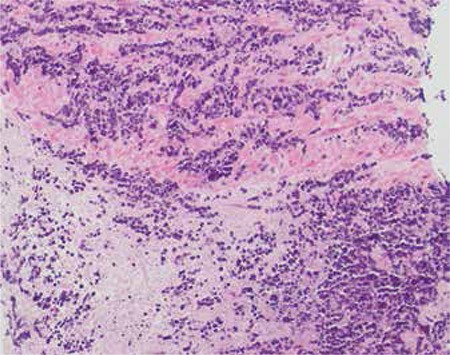
Core biopsy of the prostate: Hematoxylin and eosin stain with x100 magnification revealed small cell carcinoma cells forming diffuse solid sheets with infiltration of prostatic muscle fibers and narrow cords with some areas of necrosis. Tumor cells demonstrated mild to moderate pleomorphism with high nuclear to cytoplasmic ratio, crowded round to irregular nuclei containing chromatin. There was no acinus formation or any component of adenocarcinoma, urothelial carcinoma, squamous cell carcinoma or sarcomatoid carcinoma. Core biopsy of the left and right seminal vesicles demonstrated identical findings (not shown)

A 79 year-old male was admitted with acute renal failure with a history of radical radiotherapy for prostate adenocarcinoma 13 years ago. The prostate specific antigen level was not elevated. The abdominopelvic CT scan showed markedly enlarged seminal vesicles occupying most of the pelvic cavity causing bilateral ureteral obstruction, and loss of fat plane between seminal vesicles and a mildly enlarged prostate gland indicating local invasion of the tumor. ^18^F-FDG PET/CT demonstrated intense ^18^F-FDG uptake (SUV_max_: 7.2) throughout the bilateral markedly enlarged seminal vesicles with diffuse involvement of the prostate (Figure 1). There was no primary pulmonary lesion or metastatic spread on either diagnostic CT of the chest or ^18^F-FDG PET/CT. Percutaneous core biopsies of the prostate and both seminal vesicles revealed diffuse small cell carcinoma (Figure 2). The patient was diagnosed with urogenital EPSCC, however, the origin of the tumor remained unclear. The patient succumbed to the disease prior to commencement of systemic therapy.

## LITERATURE REVIEW AND DISCUSSION

Small-cell carcinoma usually originates from the lung and accounts for 18% of lung cancers, but very rarely the primary site is detected outside the lungs and pleural spaces, which is called EPSCC (1). Small cell carcinoma of the prostate accounts for less than 1% of all prostate cancers while there are only a few case reports of primary small cell carcinoma of the seminal vesicles (2). Seminal vesicle malignancies are mostly secondary tumors from adjacent organs such as the prostate rather than primary intrinsic tumors (3). Several different routes of invasion have been suggested, most commonly from tumor extension through the ejaculatory ducts or direct spread across the prostatic base (4). Approximately 25-40% of cases is initially diagnosed as prostatic adenocarcinoma and recurs as small cell carcinoma after initial therapy, and the median interval between diagnosis of prostatic adenocarcinoma and recurrence of small cell carcinoma is reported as 25 months (2). Small cell carcinoma commonly presents with distant metastases in up to 25% of patients with small cell carcinoma of the prostate, and with locally advanced disease in some cases due to absence of symptoms in early stages (2). The microscopic features of urogenital small cell carcinoma are similar to those seen in other organs and show high grade and poor differentiation (5). The prognosis of urogenital small cell carcinoma is poor with a median survival of less than 1 year (6). Early diagnosis and accurate staging are important since surgery with curative intent may only be considered for localized disease (7). Recommended treatment regimens for urogenital small cell cancer are similar to those for small cell carcinoma of the lung. Chemotherapy is the mainstay of treatment, with radiation therapy being used either to enhance local control or for palliation of symptoms in metastatic disease (8). 18F-FDG PET imaging has been shown to be useful for staging small cell lung cancer but the rarity of urogenital small cell cancer has precluded a similar analysis (9). However, the demonstration of marked ^18^F-FDG uptake in primary small cell carcinoma of urogenital origin indicates that there may be a role for ^18^F-FDG PET/CT scan in the staging and probable response assessment of EPSCC.

## References

[1] Hindson DA, Knight LL, Ocker JM (1985). Small cell carcinoma of the prostate. Transient complete remission with chemotherapy. Urology.

[2] Wang W, Epstein JI (2008). Small cell carcinoma of the prostate. A morphologic and immunohistochemical study of 95 cases. Am J Surg Pathol.

[3] Kim B, Kawashima A, Ryu JA, Takahashi N, Hartman RP, King BF (2009). Imaging of the seminal vesicle and vas deferens. Radiographics.

[4] Sala E, Akin O, Moskowitz CS, Eisenberg HF, Kuroiwa K, Ishill NM, Rajashanker B, Scardino PT, Hricak H (2006). Endorectal MR imaging in the evaluation of seminal vesicle invasion: diagnostic accuracy and multivariate feature analysis. Radiology.

[5] Nicholson SA, Beasley MB, Brambilla E, Hasleton PS, Colby TV, Sheppard MN, Falk R, Travis WD (2002). Small cell lung carcinoma (SCLC): a clinicopathologic study of 100 cases with surgical specimens. Am J Surg Pathol.

[6] Tetu B, Ro JY, Ayala AG, Johnson DE, Logothetis CJ, Ordonez NG (1987). Small cell carcinoma of the prostate. Part I. A clinicopathologic study of 20 cases. Cancer.

[7] Bolton DM, Chiu ST, Clarke S, Angus D (1994). Primary small cell carcinoma of the prostate: unusual modes of presentation. Aust N Z J Surg.

[8] Papandreou CN, Daliani DD, Thall PF, Tu SM, Wang X, Reyes A, Troncoso P, Logothetis CJ (2002). Results of a phase II study with doxorubicin, etoposide, and cisplatin in patients with fully characterized small-cell carcinoma of the prostate. J Clin Oncol.

[9] Fischer BM, Mortensen J, Langer SW, Loft A, Berthelsen AK, Petersen BI, Daugaard G, Lassen U, Hansen HH (2007). A prospective study of PET/CT in initial staging of small-cell lung cancer: comparison with CT, bone scintigraphy and bone marrow analysis. Ann Oncol.

